# HIF1 Stabilization by Roxadustat Improves Cognition and Prevents Neuron Loss in Alzheimer’s Diseases In Vivo

**DOI:** 10.3390/biology15141118

**Published:** 2026-07-10

**Authors:** Elena V. Mitroshina, Polina L. Strelkova, Maria V. Korokozova, Maria V. Vedunova

**Affiliations:** Institute of Biology and Biomedicine, Lobachevsky State University of Nizhny Novgorod, 23 Gagarin Ave., 603022 Nizhny Novgorod, Russiamvedunova@yandex.ru (M.V.V.)

**Keywords:** Alzheimer’s disease, neurodegeneration, hypoxia, HIF-1, Roxadustat, neuroprotection

## Abstract

Alzheimer’s disease (AD) is one of the most prevalent neurodegenerative disorders worldwide and is characterized by progressive memory impairment, cognitive decline, and behavioral dysfunction. The brain’s high energy demand makes it vulnerable to hypoxia, which can trigger AD pathology. Hypoxia-inducible factor (HIF) is a transcription factor that mediates cellular and tissue adaptation to low oxygen levels. HIF-1 plays a dual role in AD: on the one hand, it is considered a potential neuroprotective target; on the other hand, its activation may exacerbate disease pathogenesis by promoting amyloid plaque formation. Given this ambiguity, further studies are needed. This study investigated the HIF prolyl hydroxylase inhibitor Roxadustat in 6-month-old male 5xFAD mice. Stabilization of the HIF-1 complex exerted a positive effect on learning ability and the retention of long-term spatial memory in 6-month-old male 5xFAD mice. Four-week treatment with Roxadustat significantly reduced pathological morphological alterations in cells of the prefrontal cortex. In addition, animals treated with Roxadustat exhibited significantly increased expression of the brain-derived neurotrophic factor (BDNF) in the cerebral cortex. Our findings suggest that stabilization of the HIF-1 complex through inhibition of HIF prolyl hydroxylase may represent a promising strategy for neuroprotection in AD.

## Simple Summary

Alzheimer’s disease is a common brain disorder that gradually impairs memory and thinking. The brain requires large amounts of oxygen to function properly, and a lack of oxygen may contribute to the development of Alzheimer’s disease. A transcription factor called hypoxia-inducible factor 1 (HIF-1) helps cells adapt to low oxygen levels, but it remains unclear whether activating this protein is beneficial or harmful in Alzheimer’s disease. To address this question, we tested a drug known as Roxadustat in mice with Alzheimer’s-like features. Roxadustat works by inhibiting HIF prolyl hydroxylase and thus stabilizing the HIF-1 complex. After four weeks of treatment, the mice showed improved learning and long-term spatial memory. Their brain cells appeared healthier, particularly in the prefrontal cortex, and levels of brain-derived neurotrophic factor (BDNF) that supports brain cell survival increased. These findings suggest that targeting this oxygen-sensing pathway may offer a new strategy for protecting the brain with Alzheimer’s disease.

## 1. Introduction

Alzheimer’s disease (AD) is the most common age-related neurodegenerative disorder, affecting millions of individuals worldwide. It is characterized by the progressive deterioration of neuronal structure and function, ultimately leading to cognitive decline and dementia [1].

Accumulating evidence indicates that AD is associated with substantial impairments in cerebrovascular function, including hypoperfusion of vulnerable brain regions, amyloid angiopathy of small vessels, and dysfunction of the neurovascular unit and the blood–brain barrier (BBB), resulting in the development of tissue hypoxia. Under hypoxic conditions, the imbalance between the production and clearance of beta-amyloid (Aβ) is exacerbated, leading to vascular and parenchymal accumulation of Aβ and the formation of amyloid plaques [2,3,4]. Hypoxia accelerates AD progression through multiple mechanisms, including increased beta-amyloid (Aβ) production, stimulation of tau protein hyperphosphorylation, induction of oxidative stress and neuroinflammation, endoplasmic reticulum (ER) stress, disruption of calcium homeostasis, and the development of excitotoxicity [5,6,7].

One of the key molecular mechanisms involved in maintaining neuronal viability under hypoxic conditions is hypoxia-inducible factor 1 (HIF-1). Members of the HIF family are heterodimeric transcription factors composed of α and β subunits. The β subunit, also known as aryl hydrocarbon receptor nuclear translocator (ARNT), is constitutively expressed and continuously present in the nucleus. In contrast, stabilization of the α subunit is regulated by cellular oxygen availability [8]. Under normoxic conditions, HIF-1α is rapidly degraded by oxygen-dependent enzymes known as prolyl hydroxylase domain proteins (PHDs). Following hydroxylation, HIF-1α is recognized by the Von Hippel-Lindau tumor suppressor (pVHL) E3 ubiquitin ligase complex and is subsequently targeted for rapid ubiquitin-dependent degradation by the 26S proteasome [9,10]. Oxygen deprivation inhibits PHD activity, resulting in the accumulation of HIF-1α, which translocates to the nucleus and dimerizes with HIF-1β to form the active HIF-1 complex.

HIF-1 regulates the expression of numerous genes whose products facilitate cellular adaptation to hypoxia. Stabilization of HIF-1 promotes efficient oxygen and nutrient delivery through induction of erythropoietin (EPO) and vascular endothelial growth factor A (VEGFA), thereby improving blood supply to brain tissue [11]. In addition, HIF-1 exerts anti-apoptotic effects and supports neuronal differentiation. HIF-1 also mediates metabolic adaptation to hypoxia by shifting cellular metabolism from oxidative phosphorylation to glycolysis. Specifically, it induces the expression of genes involved in glucose transport and glycolysis, including glucose transporters 1 and 3 (GLUT1 and GLUT3), pyruvate dehydrogenase kinase 1 (PDK1), and lactate dehydrogenase. Expression of these genes is markedly impaired in AD [6]. Moreover, several studies have demonstrated that HIF-1α levels are reduced in AD [12,13]. These observations identify HIF-1 as an attractive target for the development of novel strategies aimed at preventing the progression of neurodegenerative processes. The most common approach to enhancing HIF-1 activity involves the use of inhibitors of HIF prolyl hydroxylases, which increase intracellular HIF-1α levels and stabilize the HIF-1 complex. Experimental evidence has demonstrated beneficial effects of PHD inhibitors in both in vivo and in vitro models of AD [14,15,16]. At the same time, HIF-1 can induce increased expression of BACE1, the principal protease responsible for the amyloidogenic β-cleavage of amyloid precursor protein (APP), suggesting a potential pathogenic link between HIF-1 activation and AD progression. In addition, HIF-1 may exert both anti-inflammatory and pro-inflammatory effects in the brain, in part through direct activation of microglia and astrocytes [6,15,17].

Taken together, these findings indicate that the role of HIF-1 in the pathogenesis of AD is complex and multifaceted and warrants further investigation.

In the present study, we investigated the effects of chronic administration of the small-molecule Roxadustat (FG-4592) inhibitor of prolyl hydroxylase domain (PHD) enzymes on the progression of neurodegenerative processes in 6-month-old 5xFAD mice. Roxadustat is a novel therapeutic agent for the treatment of anemia in patients with chronic kidney disease [8], acting as an inhibitor of HIF-prolyl hydroxylase 1 (PHD1). HIF-prolyl hydroxylases are α-ketoglutarate- and Fe^2+^-dependent dioxygenases. Roxadustat is a low-molecular-weight analog of α-ketoglutarate and inhibits HIF-prolyl hydroxylase through competitive binding to the catalytic center of the enzyme [18]. Its ability to partially cross the blood–brain barrier and induce HIF-1α expression in brain tissue [19] makes it a promising candidate for investigating potential neuroprotective properties. Ongoing studies are investigating its potential use in the therapy of ischemic stroke [20,21], hypoxic brain injury [22,23], neurodegenerative diseases [19], and other conditions. We assessed changes in neurological status, memory, and cognitive function, as well as the expression levels of neurotrophic factors and pro-inflammatory cytokines and morphological alterations in the prefrontal cortex of 5xFAD mice following a 4-week course of Roxadustat administration. Roxadustat treatment was shown to exert a neuroprotective effect.

## 2. Materials and Methods

### 2.1. Subject of the Study

The study was conducted using 6-month-old 5xFAD male mice and wild-type (WT) mice (n = 29). 5xFAD mice carry five mutations in the APP and PSEN1 transgenes (APP: Swedish K670N/M671L, Florida I716V, and London V717I; PSEN1: M146L and L286V) and are characterized by the rapid development of amyloid pathology and cognitive impairment beginning at 4 months of age [24,25]. Animal housing and experimental procedures were performed in accordance with the Rules for Carrying Out Work Using Experimental Animals (Russia, 2010), the International Guiding Principles for Biomedical Research Involving Animals (CIOMS and ICLAS, 2012), and the ethical principles set forth in the European Convention for the Protection of Vertebrate Animals Used for Experimental and Other Scientific Purposes (Strasbourg, 2006). All experimental procedures were approved by the Bioethics Commission of Lobachevsky State University of Nizhny Novgorod (Protocol No. 61, 24 January 2023).

The animals were randomly assigned to three groups:

- Wild-type (WT) mice (n = 10);
- 5xFAD mice serving as the control group and receiving no drug treatment (n = 10);
- 5xFAD mice treated with Roxadustat (MedChemExpress, Monmouth Junction, NJ, USA) by intraperitoneal injection at a dose of 10 mg/kg once every three days for 4 weeks (n = 9).

The animals were housed in the SPF vivarium of Lobachevsky State University of Nizhny Novgorod, which possesses a valid veterinary license for the maintenance, breeding, and sale of laboratory animals (Certificate No. 52-005921, dated 1 July 2022). The animals had unrestricted access to food and water, except during designated experimental procedures. The light/dark cycle in the vivarium was maintained at 12/12 h. To exclude the influence of the order of tests and manipulations, the animals were tested in random order. To eliminate the influence of circadian rhythms, all experiments were conducted at the same time of day.

Sample sizes for in vivo experiments were determined using G*Power 3.1.5 software. All mice were randomly allocated for the treatment. Everyone involved in the experiments and analyses of their outcomes was blinded. An investigator, blinded to the identity of each assessment group, conducted the neurological evaluations and behavioral tests. To minimize potential confounding factors, all animals were introduced into the experiment simultaneously and were kept under standard conditions.

### 2.2. Assessment of Neurological Status

The functional state of the nervous system was evaluated weekly using the neurological deficit severity scale for small laboratory animals. Neurological status was assessed weekly during drug administration and for two weeks after the completion of treatment. The assessment comprised 13 tests evaluating the following parameters: the ability of the mouse to reach toward a surface with both forepaws while suspended above it, both with and without contact of the vibrissae with the surface (at suspension heights of 50 cm and 1.5–2 m); the ability to maintain balance on a 10-mm-diameter rod for 10 s; restoration of posture following lateral displacement of the body; ability to move in a straight line; the presence of ptosis, exophthalmos, tail curvature, and the paw withdrawal reflex in response to pinprick stimulation; preservation of the grasping reflex; the degree of lordosis and kyphosis; and the ability and initiative to leave a 30-cm-diameter circle within 3 min. Each test was scored as follows: 0 points for successful performance, 1 point for partial performance, and 2 points for failure to perform the task. In cases where a particular sign was expressed asymmetrically, a score of 0.5 points was assigned [23,26,27]. The individual scores were subsequently summed. Neurological status was interpreted according to the following criteria:

- 10–17 points: Severe CNS impairment;
- 5–9 points: Moderate CNS impairment;
- 1–4 points: Mild CNS impairment.

### 2.3. Open Field Test

Open field testing was performed prior to drug administration (Week 0) and subsequently once every two weeks for 6 weeks to assess locomotor and exploratory activity. The procedure was conducted using a specialized apparatus (OpenFieldL E800S; Panlab Harvard Apparatus, Cornella de Llobregat, Spain), consisting of a square arena enclosed by high walls and covered with a sensor-equipped frame. Each animal was placed in the center of the arena for 5 min. The total distance traveled, average locomotor speed, time spent in the central and peripheral zones of the arena, number of rearing events, and freezing time were recorded. Data analysis was performed using Smart v. 3.0.03 software (Panlab Harvard Apparatus, Cornella, Spain; Stoelting, Wood Dale, IL, USA) [27].

### 2.4. Conditioned Passive Avoidance Reflex (CPA) Test

The CPA test was performed using a chamber consisting of illuminated and dark compartments separated by a partition with a door (Shuttle Box LE918; Panlab Harvard Apparatus, Cornella, Spain). Prior to Roxadustat administration, animals underwent a single training session. Mice were placed in the illuminated compartment, and the latency to enter the dark compartment was recorded. Upon entry into the dark compartment, an electric current (0.3–0.6 mA for 5 s) was delivered through the grid floor, inducing moderate nociceptive stimulation, after which the animals were returned to their home cages.

Testing was performed 24 h after training and subsequently repeated twice: immediately after completion of the Roxadustat administration course and two weeks after treatment completion. During testing, the same chamber was used without application of electric current. The latency to enter the dark compartment was recorded over a 180-s observation period [23].

### 2.5. Morris Water Maze Test

The Morris water maze test was used to assess long-term spatial memory and learning in laboratory animals. The apparatus consisted of a cylindrical pool filled with opaque water maintained at 23–25 °C. A platform was positioned within the pool, with its surface submerged 0.5–1 cm below the water level. In addition, an external visual cue (a black triangle) was attached to the side wall of the pool. Training in the water maze was conducted over five consecutive days following completion of the drug administration course. During each training session, the animals performed three trials to locate the hidden platform, with each trial lasting 60 s. The mice were introduced into the water from three different starting points located in separate sectors of the pool. Beginning on the second day of training, the latency to locate the platform was recorded for each trial. An unsuccessful attempt during water maze training was defined as failure to find the hidden platform within 60 s. In such cases, mice were guided to the platform and allowed to remain on it for 15 s. The three daily trials were averaged to obtain a mean daily latency per mouse. The water was changed daily, and the maze was thoroughly mixed between each trial. To control for odor cues, the water was stirred between trials. Retention of the memory trace was evaluated 14 days after the final training session. During this testing phase, the platform was removed from the pool. The time spent searching in the target quadrant and the time spent in the area previously occupied by the platform were assessed [27]. Platform search strategies were also analyzed. Three distinct search strategies were identified: direct search, in which the mouse moved purposefully toward the former platform location; active search, characterized by circular movements along the periphery with rapid correction of direction when deviating from the target; and chaotic search, characterized by the absence of a defined strategy and frequent changes in movement direction [26,27].

### 2.6. Brain Morphology Assessment

For histological analysis, brain tissue was fixed in 4% paraformaldehyde for 24 h at 25 °C. Subsequently, the samples were dehydrated through a graded series of alcohols and xylene in the following sequence: 50% isopropyl alcohol (IPA) for 1 h, followed by sequential incubation in increasing concentrations of alcohol: 70% IPA (1 h), 80% IPA (17 h), 96% IPA 1 (30 min), and 96% IPA 2 (30 min). This was followed by incubation in intermediate media: IPA + xylene (30 min at 37 °C), xylene (30 min), and xylene + paraffin (30 min). The samples were then embedded in paraffin (Biovitrum, Saint-Petersburg, Russia) and incubated for 1 h at 57–58 °C. Sections of 3 µm thickness were cut using a Thermo Fisher Scientific HM355S microtome (Thermo Fisher Scientific, Bremen, Germany).

Subsequently, sections were deparaffinized by passage through xylene and rehydrated in graded alcohols. Staining was performed with Mayer’s hematoxylin (OOO “ErgoProduction”, BioVitrum, Saint-Petersburg, Russia) for 10 min and with 1% aqueous eosin (Leica, Deerfield, IL, USA) for 1 min. Specimens were visualized using a Zeiss Primo Star microscope (Zeiss, Jena, Germany) equipped with an integrated Axio CamMRc camera (Zeiss, Jena, Germany). Acquired images were analyzed using the open source software ImageJ 1.54k. Three sections of the prefrontal cortex were obtained from each animal. Ten fields of view within the prefrontal cortex were evaluated for each section [27]. The parameter values from the ten fields were averaged as technical replicates, and each mouse was considered a biological replicate. The following parameters were assessed: total number of neurons and glial cells per field of view; neuronal soma diameter; nuclear diameter; perikaryal cytoplasmic thickness; nuclear to cytoplasmic ratio; the number of non-shrunken and shrunken hyperchromatic neurons per field of view; the number of necrotic and apoptotic elements; the number of ghost cells; the number of cells with pericellular edema; and the extent of edema.

### 2.7. Real-Time Polymerase Chain Reaction

The expression levels of HIF-1α, the neurotrophic factors BDNF and GDNF, the pro-inflammatory cytokines IL-1β, IL-6, and TNF-α, as well as APP, were evaluated in the prefrontal cortex of the experimental animals using quantitative real-time polymerase chain reaction (qRT-PCR). Total RNA was isolated using the RNA-Extran kit (Syntol, Moscow, Russia) according to the manufacturer’s instructions. RNA concentration was determined by UV spectrophotometry using a NanoDrop One spectrophotometer (Thermo Fisher Scientific, Waltham, MA, USA). Complementary DNA (cDNA) synthesis was performed by reverse transcription using the RT-M-MuLV-RH kit (Biolabmix, Novosibirsk, Russia) in accordance with the manufacturer’s protocol. Quantitative real-time PCR was carried out using the BioMaster HS-qPCR SYBR Blue (2×) kit (Biolabmix, Novosibirsk, Russia). The primer sequences used in the study are presented in Table 1.

Amplification was performed using the following protocol: initial denaturation at 95 °C for 5 min, followed by 40 cycles consisting of 95 °C for 20 s, 60 °C for 20 s, and 72 °C for 20 s, using a QuantStudio real-time PCR system (Thermo Fisher Scientific, Waltham, MA, USA). Oaz1 was used as the reference gene. The data were normalized to the “WT” group and analyzed using the ΔΔCt method [26,28].

### 2.8. Statistical Analysis

The data are presented as mean (M) ± standard error of the mean (SEM). One-way and two-way analyses of variance (ANOVA) followed by Tukey’s multiple comparisons test were used for statistical analysis. For nonparametric data, the Kruskal–Wallis test followed by Dunn’s post hoc test or the Mann–Whitney U test was applied. For longitudinal data a two-way mixed-design ANOVA was used, with treatment as the between-subjects factor and time as the within-subjects factor. Post hoc comparisons were performed using Tukey’s HSD test. Statistical analysis was performed using GraphPad Prism software, version 8.0. Differences were considered statistically significant at *p* ≤ 0.05.

## 3. Results

### 3.1. Effect of Chronic Roxadustat Administration on the Progression of Neurological Deficits and Motor Activity in 5xFAD Mice

**Figure 1:**
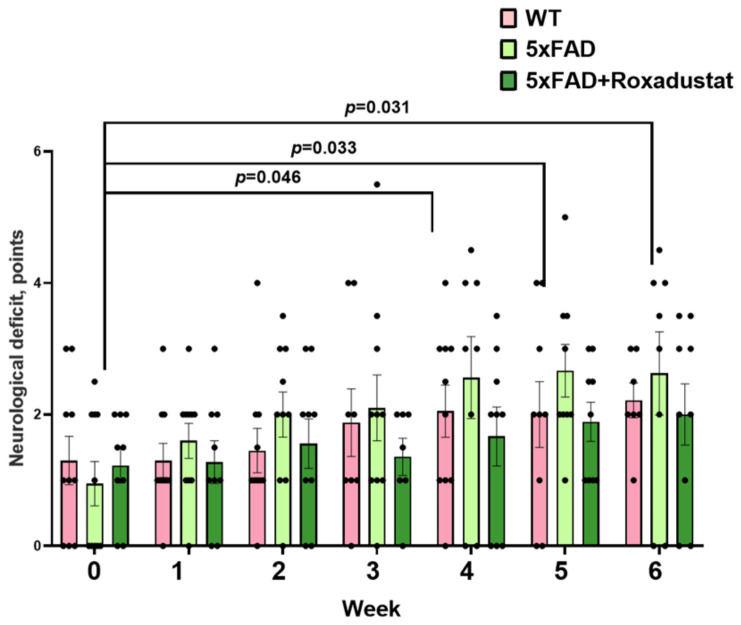
Dynamics of neurological status 5xFAD mice during Roxadustat treatment. The black dots in the graphs represent individual data points. Differences were considered significant at *p* ≤ 0.05 (Wilcoxon test).

Analysis of the dynamics of neurological status over the six-week observation period demonstrated that the condition of wild-type animals remained stable throughout the study. In the “5xFAD” group, a progressive and significant deterioration in neurological status was observed, reaching statistically significant differences compared by Week 4 of the experiment (Figure 1). In contrast, animals in the “5xFAD + Roxadustat” group exhibited only mild CNS impairment throughout the entire observation period, with no significant differences compared with the “WT” group. Notably, by Week 6 of the experiment, two animals in the “5xFAD + Roxadustat” group exhibited no signs of neurological deficit (score = 0).

**Figure 2:**
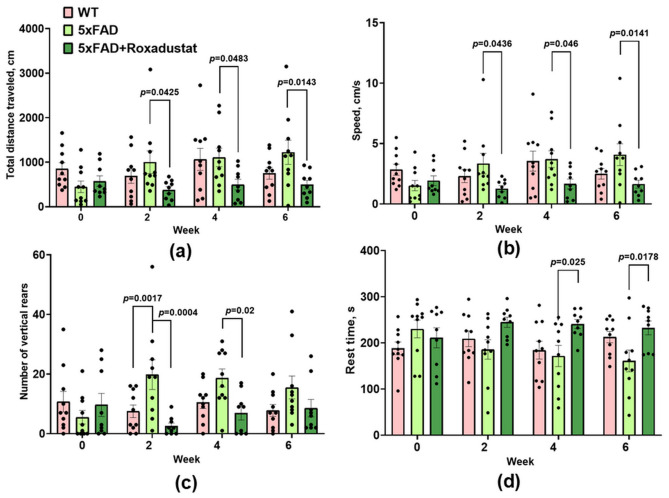
Key parameters of locomotor and exploratory activity in 5xFAD mice during Roxadustat treatment in the “Open Field” test: (**a**) total distance traveled; (**b**) average locomotor speed; (**c**) number of vertical rearings; (**d**) freezing time. The black dots in the graphs represent individual data points. Differences were considered significant at *p* ≤ 0.05 (ANOVA).

The locomotor and exploratory activity of wild-type animals remained unchanged throughout the experiment. Interestingly, animals in the “5xFAD” group demonstrated increased locomotor and exploratory activity beginning from Week 3 of the experiment. This was manifested by a statistically significant increase in the number of vertical rearings (Week 1: 5.5 ± 2.27; Week 3: 19.8 ± 4.9; Week 5: 19.8 ± 4.9), as well as a persistent tendency toward an increase in total distance traveled and average locomotor speed within the arena, accompanied by a reduction in freezing time (Figure 2).

Analysis of behavioral responses following administration of the HIF-prolyl hydroxylase inhibitor demonstrated that, beginning from Week 3 of the experiment, locomotor activity in the experimental group was significantly lower than that observed in the “5xFAD” group and did not differ from the parameters recorded in the “WT” group (total distance, Week 7: “5xFAD” 1223.3 ± 270.8 cm vs. “5xFAD + Roxadustat” 494.9 ± 98.2 cm; average speed, Week 7: “5xFAD” 4.1 ± 0.9 cm/s vs. “5xFAD + Roxadustat” 1.6 ± 0.3 cm/s). A reduction in the number of vertical rearings was also observed during Weeks 3 and 5 of the experiment—by 7.8-fold and 2.7-fold, respectively—compared with the “5xFAD” group; however, by Week 7, these differences were no longer observed (Figure 2c). It is important to note that the behavior of mice in the “5xFAD + Roxadustat” group was characterized by increased anxiety-like behavior, as reflected by prolonged freezing time during Weeks 5 and 7 of the experiment (Figure 2d). The remaining parameters assessed in the Open Field test showed no statistically significant differences between groups after 4 weeks of drug administration.

Thus, prolonged stabilization of HIF-1 slowed the progression of neurological deficits in 5xFAD mice; however, their behavioral responses were characterized by increased anxiety-like behavior.

### 3.2. Effect of Chronic Roxadustat Administration on Cognitive Function and Memory in 5xFAD Mice

**Figure 3:**
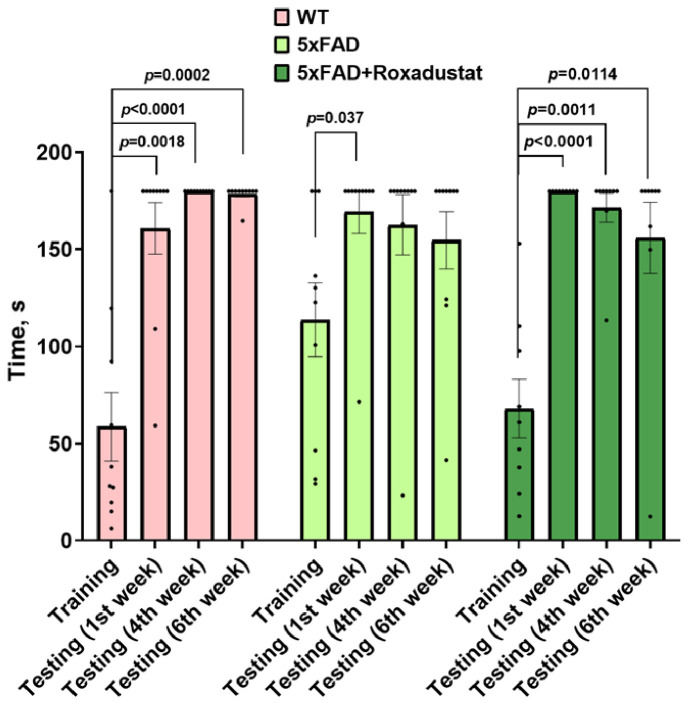
Passive avoidance test. Latency to enter the dark compartment during conditioned passive avoidance reflex formation. The black dots in the graphs represent individual data points. Differences were considered significant at *p* ≤ 0.05 (Wilcoxon test).

Assessment of memory using the passive avoidance test demonstrated that, when tested 24 h after training (prior to the initiation of drug administration), all animals retained the memory trace, as evidenced by a significantly prolonged latency to enter the dark compartment compared with the training session. During subsequent re-testing, the latency period in animals of the “5xFAD” group did not differ significantly from the values recorded during training, indicating extinction of the memory trace. One month after completion of PHD inhibitor administration, the majority of wild-type mice (90%) continued to retain the memory trace and did not enter the dark compartment of the apparatus. In contrast, 31.6% of 5xFAD mice entered the dark compartment during the testing period, which may indicate the development of mnemonic impairment (Figure 3). In the “5xFAD + Roxadustat” group, a significant increase in latency to enter the dark compartment, relative to the training session, was maintained throughout the entire observation period, although entry into the dark compartment was recorded in 33% of the animals.

**Figure 4:**
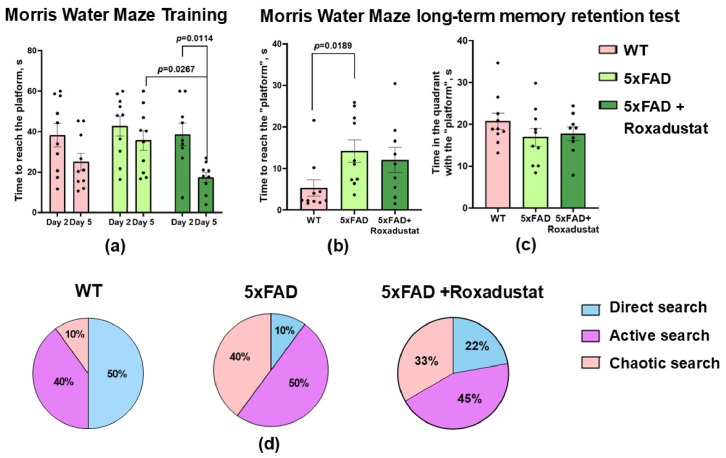
Key parameters assessed in the Morris water maze test: (**a**) latency to locate the platform on Days 2 and 5 of training; (**b**) time spent searching the platform; (**c**) time spent in the zone previously occupied by the platform; (**d**) platform search strategies. The black dots in the graphs represent individual data points. Differences were considered significant at *p* ≤ 0.05 (Kruskal–Wallis test).

We also evaluated the animals’ capacity for navigational learning and long-term spatial memory formation using the Morris water maze test. Animals in the “5xFAD + Roxadustat” group demonstrated superior learning ability compared with the other experimental groups, as evidenced by a statistically significant reduction in the latency to locate the platform during the fifth training session (Figure 4a). It is noteworthy that, in the “5xFAD + Roxadustat” group, 22% of animals failed to locate the hidden platform in one of the three trials on Day 5 of training, whereas in the “5xFAD” group, unsuccessful attempts were observed in 40% of animals. These findings indicate that HIF-1 stabilization improves cognitive function and learning ability under conditions of developing neurodegenerative processes.

Assessment of memory trace retention two weeks after training revealed that animals in the “5xFAD” group spent significantly more time searching the target quadrant compared with the “WT” group, confirming the development of mnemonic impairment (Figure 4b). In contrast, the time spent searching the target quadrant in the “5xFAD + Roxadustat” group did not differ from that observed in wild-type animals (Figure 4b). No significant differences in platform search strategies were detected (Figure 4d).

Thus, the obtained data demonstrate that chronic administration of a HIF-prolyl hydroxylase inhibitor improved learning ability and partially prevented long-term memory impairment in the experimental animals.

### 3.3. Effect of Chronic Roxadustat Administration on the mRNA Expression of Pro-Inflammatory Cytokines and Neurotrophic Factors

To investigate the effect of HIF-1 complex stabilization on the development of neuroinflammation and the expression of neurotrophic factors in Alzheimer’s disease, RT-PCR analysis was performed to determine the mRNA expression levels of BDNF, GDNF, IL-1β, IL-6, TNF-α, and APP in the prefrontal cortex. In addition, the mRNA expression level of HIF-1α was evaluated.

**Figure 5:**
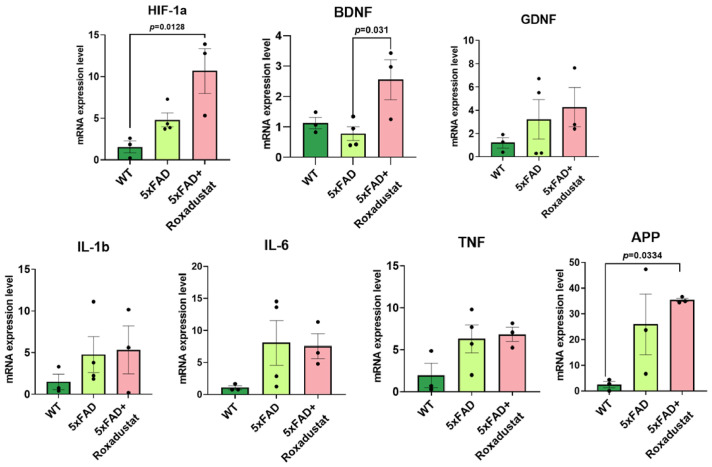
Relative mRNA expression levels in the cerebral cortex 5xFAD mice after chronic Roxadustat treatment. Data are normalized relative to the “WT” group The black dots in the graphs represent individual data points. Differences were considered significant at *p* ≤ 0.05 (Kruskal–Wallis test).

The results demonstrated that administration of Roxadustat, a HIF-prolyl hydroxylase inhibitor, significantly increased HIF-1α mRNA expression relative to the “WT” group. Compared with the “5xFAD” group, HIF-1α mRNA expression increased 2.73-fold (Figure 5). We also observed a significant 2.1-fold increase in BDNF mRNA expression in the cerebral cortex of mice treated with Roxadustat compared with the “5xFAD” group. APP mRNA expression in the “5xFAD + Roxadustat” group was significantly elevated relative to wild-type animals but did not differ from the levels observed in the “5xFAD” group.

No significant effects of Roxadustat treatment were observed with respect to the expression levels of the major pro-inflammatory cytokines IL-1β, IL-6, and TNF-α (Figure 5).

### 3.4. Histological Changes in the Prefrontal Cortex

**Figure 6:**
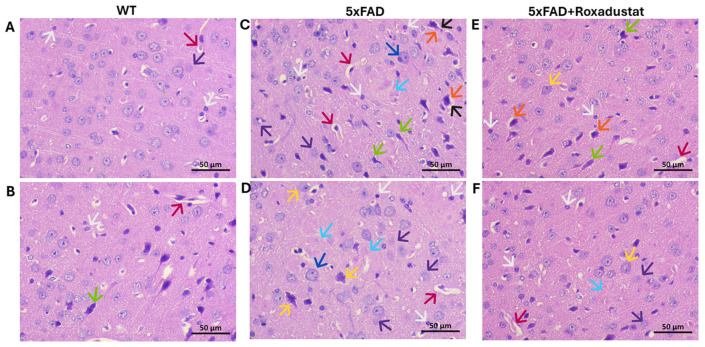
Representative photomicrographs of hematoxylin and eosin-stained histological sections of the prefrontal cortex of mouse cerebral hemispheres. (**A**,**B**) “WT”; (**C**,**D**) “5xFAD”; (**E**,**F**) “5xFAD + Roxadustat”. Blue arrows indicate swollen cells; orange arrows indicate pericellular edema; green arrows indicate shrunken hyperchromatic neurons; black arrows indicate non-shrunken hyperchromatic neurons; yellow arrows indicate cells undergoing apoptosis or necrosis; purple arrows indicate ghost cells; light blue arrows indicate focal neuronal loss; pink arrows indicate perivascular edema; white arrows indicate glial cells. Scale bar: 50 µm.

Histological analysis of the prefrontal cortex was performed one month after completion of the Roxadustat treatment course. At the time of histological examination, the animals were 8 months old. It was demonstrated that neurons in animals of the “WT” group exhibited normal morphology, approximating a rounded or pyramidal shape, with a mean neuronal soma diameter of 12.19 ± 0.23 µm (Figure 6A,B; Table 2). The cytoplasm was normochromic, whereas the nuclei were rounded and uniformly filled with chromatin, containing a predominantly centrally located nucleolus with distinct and smooth contours. The cells were arranged in an orderly manner, with a mean of 61.04 ± 2.31 neurons per field of view. Occasional neurons with signs of pericellular edema were observed (3.2 ± 0.52 cells per field of view), along with hyperchromatic neurons of preserved morphology (non-shrunken neurons) and shrunken hyperchromatic neurons, numbering 6.7 ± 0.77 and 3.76 ± 0.55 cells per field of view, respectively. Blood vessels were characterized by thickened walls, and endothelial cells were tightly apposed to one another.

In the “5xFAD” group, alongside morphologically normal neurons, cells with irregular and indistinct contours, abnormal morphology, and disordered arrangement were observed (Figure 6C,D). The diameter of the neuronal perikaryon was increased, reaching 13.82 ± 0.3 µm, while the nuclei were also enlarged (10.56 ± 0.21 µm) and occupied more than half of the cytoplasmic volume (Table 2). Both reversible and irreversible cellular alterations characteristic of different stages of degeneration were identified. The number of hyperchromatic neurons per field of view, including both shrunken and non-shrunken forms, was increased by 12.78% compared with the WT mice (Table 2). Cells exhibiting signs of apoptosis and necrosis were observed (3.24 ± 0.23 per field of view), as well as “ghost cells” (3.96 ± 0.21 per field of view), characterized by the absence of a nucleus, nucleolus, and distinct cellular contours. In addition, the numbers of neurons exhibiting signs of swelling (1.73 ± 0.17 per field of view), pericellular edema (5.11 ± 0.71 per field of view), and cytoplasmic vacuolization (1.23 ± 0.22 per field of view) were significantly increased. Areas of complete focal neuronal loss were also identified, with an average size of 34.58 × 31.54 µm.

Most blood vessels exhibited narrowed lumens or appeared collapsed; however, congested vessels were also observed. The perivascular space was enlarged by 26.3% compared with the “WT” group, reaching 3.84 ± 0.12 µm, and the vascular walls appeared thinned.

Administration of Roxadustat significantly improved the morphological state of the prefrontal cortex. A substantial proportion of cells retained normal morphology. The nuclei were round or oval and centrally localized within the neuronal cell bodies. In addition, neuronal organization appeared more structured (Figure 6E,F). The mean cell diameter was 12.67 ± 0.22 µm, which differed only minimally from that observed in the WT group. In the “5xFAD + Roxadustat” group, the number of cells exhibiting signs of necrosis and apoptosis was reduced by 39.5% compared with the “5xFAD” group, reaching 1.96 ± 0.19 cells per field of view. A reduction was also observed in the number of ghost cells (by 37.12%, to 2.49 ± 0.18 cells per field of view) and cells exhibiting signs of swelling (by 49.71%, to 0.87 ± 0.14 cells per field of view); these values were significantly lower than those in the “5xFAD” group. Areas of focal neuronal loss were significantly smaller than in the “5xFAD” group, with a mean size of 28.26 × 24.78 µm, and occurred 1.9-fold less frequently. A trend toward reduction was also observed in the number of non-shrunken (4.62 ± 0.65) and shrunken (4.07 ± 0.55) hyperchromatic neurons, as well as in cells exhibiting pericellular edema and cytoplasmic vacuolization. Perivascular edema in the “5xFAD + Roxadustat” group was less pronounced; engorged blood vessels were observed, and the number of newly formed vessels appeared increased.

Thus, treatment with the HIF-prolyl hydroxylase inhibitor Roxadustat at a dose of 10 mg/kg every three days for four weeks reduced pathological morphological alterations in the prefrontal cortex of animals with an experimental model of Alzheimer’s disease. This effect was manifested by decreased proportion of damaged cells, and reduced neuronal death. These findings suggest that stabilization of HIF-1 exerts a pronounced neuroprotective effect.

## 4. Discussion

Chronic hypoxia is considered one of the major predisposing factors in the pathogenesis of AD [29]. A number of comorbid conditions, including chronic obstructive pulmonary disease (COPD), obstructive sleep apnea syndrome (OSAS), and various vascular disorders associated with chronic hypoxia, have been linked to an increased risk of AD [2,30,31]. Cerebral amyloid angiopathy is a common pathological feature of AD and is characterized by extracellular deposition of Aβ within the walls of cerebral blood vessels, particularly capillaries [2]. Hypoxia activates multiple metabolic cascades that contribute to the development of neurodegeneration. Oxidative stress induced by hypoxic injury increases the activity and expression of γ-secretase and β-secretase (BACE1), thereby enhancing the production of neurotoxic Aβ1–42 species. In addition, hypoxia markedly decreases the expression and activity of the metalloproteinases ADAM10 and ADAM17—the principal enzymes responsible for non-amyloidogenic cleavage of APP—as well as the expression of neprilysin (NEP), a key enzyme involved in amyloid clearance in neuronal cells [6,7]. Aβ production and hypoxia-induced oxidative stress mutually potentiate one another. A similar relationship has been described for tau protein, since chronic hypoxia may activate the protein kinases GSK-3β and CDK5 while inhibiting the phosphatase PP2A, thereby promoting tau hyperphosphorylation and the formation of neurofibrillary tangles [32,33]. Accumulation of Aβ disrupts neuronal calcium homeostasis, and elevated intracellular Ca^2+^ levels may subsequently induce excitotoxic cell death. Increased cytosolic Ca^2+^ concentrations may further stimulate Aβ production, thus establishing a vicious cycle of neurodegeneration that ultimately results in progressive cognitive decline in patients with AD [5,7].

Accordingly, the development of strategies aimed at enhancing the adaptive capacity of neuronal cells under conditions of chronic hypoxic injury appears highly promising. One of the principal mechanisms underlying the cellular response to oxygen deprivation is the activation and stabilization of the transcription factor HIF-1. PHD inhibitors are widely used to modulate HIF activity. One of the first PHD inhibitors introduced into clinical practice was the small-molecule inhibitor FG-4592 (Roxadustat), a 2-oxoglutarate analog that induces transient activation of HIF signaling. Developed by FibroGen in collaboration with Astellas/AstraZeneca, Roxadustat was approved in China in December 2018 and in Japan in September 2019 for the treatment of anemia in dialysis-dependent patients with chronic kidney disease [8]. Several studies have demonstrated that Roxadustat partially penetrates the blood–brain barrier and induces HIF-1α expression in brain tissue, making it a promising candidate for the treatment of neurological disorders. Neuroprotective effects of Roxadustat have been demonstrated in several in vivo studies using mouse models of Parkinson’s disease and acute neuroinflammation [19,34]. However, to date, no data are available regarding the effects of this PHD inhibitor on the progression of Alzheimer’s disease.

In the present study, we evaluated the effects of a four-week course of Roxadustat administration (10 mg/kg) on the progression of neurodegenerative processes in 5xFAD mice, a widely used transgenic model of AD. Stabilization of HIF-1 effectively prevented the progression of neurological deficits in 5xFAD mice and improved learning performance in the Morris water maze test. However, it did not significantly affect memory retention, which may be attributable to the prolonged interval between completion of treatment and delayed behavioral testing.

Histological analysis revealed that morphological alterations in neurons of the prefrontal cortex in mice of the “5xFAD” group were characterized by a tendency toward the development of structural and metabolic dysfunction. These alterations were manifested by changes in cellular morphology and an increased number of damaged cells, including swollen neurons, cells exhibiting pericellular edema, and shrunken hyperchromatic neurons, as well as pronounced signs of apoptosis and, to a lesser extent, necrosis. Subsequently, these cells transformed into ghost cells, ultimately resulting in neuronal dropout and cell death. As a consequence of neuronal loss, one of the characteristic features of the aging brain—cellular rarefaction—was observed, as evidenced by focal areas of complete neuronal loss.

The vascular alterations and changes in vessel wall morphology identified in the “5xFAD” group further support the hypothesis that impaired cerebral blood supply contributes to the pathogenesis of AD. An increased rate of cell death via both apoptotic and necrotic pathways was observed. In turn, reduced neuronal density in the cerebral cortex may contribute to impairments in learning and memory. Moreover, these pathological changes in neural tissue were accompanied by an inflammatory response, as evidenced by the development of tissue edema and an increased number of glial cells. Administration of Roxadustat, an inhibitor of HIF-prolyl hydroxylase, resulted in a significant reduction in pathological morphological alterations in the “5xFAD + Roxadustat” group. In particular, reduction in the number of damaged and dead cells were observed. The prefrontal cortex and its interaction with the hippocampus play a critical role in spatial navigation, particularly in the implementation of effective goal-directed strategies and behavioral flexibility [35]. PFC damage, including that associated with Alzheimer’s disease, impairs the execution of navigational strategies [36]. Moreover, the PFC is critically involved in working and short-term spatial memory, deficits of which emerge already at preclinical stages of Alzheimer’s disease [37]. In particular, early PFC-dependent spatial memory deficits associated with hyperactivity of prefrontal parvalbumin interneurons have been described in 5xFAD mice [38]. Thus, the morphological and molecular changes observed in the PFC reflect the preservation of a key node within the hippocampal–prefrontal navigation circuit, which substantiates the link between these changes and the behavioral effects.

To clarify the potential mechanisms underlying the neuroprotective effects of Roxadustat, we analyzed the mRNA expression levels of several genes of interest. We hypothesize that the neuroprotective effect of chronic Roxadustat administration is mediated by stabilization of the transcription factor HIF-1. Our results demonstrated that treatment with Roxadustat significantly increased HIF-1α mRNA expression. Although we did not directly quantify HIF-1α protein levels, numerous studies confirm that Roxadustat can increase HIF-1α protein levels in brain tissue. For instance, in [39] showed that Roxadustat potently stabilizes HIF-1 and, unlike the PHD inhibitor DMOG, can cross the blood–brain barrier. Yan and colleagues demonstrated a dose- and time-dependent increase in HIF-1α in PC12 cells and in the striatum, prefrontal cortex, and hippocampus of mice following Roxadustat administration at 10 mg/kg [40]. Similar results were obtained in an in vivo model of migraine, where Roxadustat increased HIF-1α protein levels and modulated the inflammatory response via the HIF-1α/NF-κB axis [41]. These data confirm that Roxadustat is an effective pharmacological tool for stabilizing HIF-1α in the CNS. Under these conditions, APP mRNA expression was elevated relative to the WT group; however, no increase was observed compared with the “5xFAD” group. Overall, HIF-1 is capable of regulating the expression of a broad range of genes directly involved in the development of amyloidosis in AD. For example, the promoter region of the BACE1 gene contains a functional hypoxia-response element (HRE), and HIF-1 regulates BACE1 expression through binding to this HRE. The γ-secretase complex consists of four proteins: presenilin 1 or 2 (PS1 or PS2), nicastrin (NCT), APH-1 (anterior pharynx defective 1), and presenilin enhancer 2 (PEN2). The promoter region of the APH-1A gene also contains HIF-1 binding sites, and APH-1 expression increases under hypoxic conditions [2,7,42]. Therefore, future studies should determine whether Roxadustat shifts APP processing toward the amyloidogenic or non-amyloidogenic pathway.

It is well-established that AD is accompanied by the development of chronic neuroinflammation, which plays a major role in disease progression [6,43]. During AD progression, Aβ accumulation and amyloid plaque formation act as DAMPs, thereby activating microglia and astrocytes [3,44]. Persistent inflammation eventually promotes the transition of microglia toward an activated pro-inflammatory M1 phenotype, leading to increased production of pro-inflammatory cytokines, including IFN-γ, IL-1β, and TNF-α. These cytokines suppress the phagocytic activity of microglia and inhibit the production of Aβ-degrading protease [45,46,47]. Similarly, pathological astrogliosis develops over time and is characterized by cellular hypertrophy and excessive release of neurotoxic and pro-inflammatory mediators [48,49]. Collectively, these processes create a chronic neuroinflammatory environment and exacerbate neuronal and synaptic loss, thereby further accelerating the progression of AD [43]. In the present study, a trend toward increased mRNA expression of the pro-inflammatory cytokines IL-1β, IL-6, and TNF-α was observed; however, these changes did not reach statistical significance. It is likely that, at 8 months of age, 5xFAD mice are transitioning from the early to the late stages of neurodegeneration, as supported by the histological findings, and that neuroinflammatory activity will continue to increase. Notably, Roxadustat administration did not significantly affect the expression of the pro-inflammatory cytokines analyzed in the present study. The absence of significant changes in IL-1β, IL-6, and TNF-α mRNA expression limits our ability to draw definitive conclusions about the anti-inflammatory effects of Roxadustat. Additional studies assessing glial cell activation (microglia and astrocytes) and other inflammatory markers, such as NFKB, IL-10, TGF-β, as well as analysis of cytokine protein levels, are required to substantiate the hypothesis that Roxadustat modulates neuroinflammatory processes.

Another important aspect examined in the present study was the potential influence of HIF-1 on the neurotrophic factors BDNF and GDNF. These neurotrophic factors are critically involved in the initiation and regulation of adult neurogenesis and also play a key role in synaptic plasticity, a process closely associated with learning and memory functions [50,51]. Moreover, reduced expression levels of these factors have been reported in patients with AD [50,51,52,53,54]. In a study by Xue et al. [55], it was proposed that the HIF-1α–EPO and cAMP–CREB–BDNF signaling pathways form a synergistic network mediating neuroprotective effects. In addition, Zhao et al. [56] described the existence of a BDNF/TrkB/HIF-1α/BNIP3 signaling axis in the context of diabetic cerebral ischemia. In the present study, we sought to evaluate the potential interaction between the BDNF- and HIF-1-related molecular pathways in the context of AD. Our results demonstrated that HIF-1α stabilization is associated with increased Bdnf mRNA expression, which correlates with the observed reduction in neuronal cell death and preservation of the normal morphological architecture of the prefrontal cortex in 5xFAD mice. It is well established that BDNF levels may correlate with disease severity [57]; thus, normalization of BDNF expression may contribute to the maintenance of the morphofunctional integrity of neuronal cells. However, we acknowledge that increased Bdnf mRNA is only correlative and does not prove a causal role for BDNF in the neuroprotective effects of Roxadustat. To establish a mechanistic link, further studies are required, for example using BDNF-neutralizing antibodies or conditional *Bdnf* knockout in specific neuronal populations.

An important limitation of the present study is the absence of direct measurement of HIF-1α protein stabilization. Although we observed an increase in Hif1a mRNA, which indirectly suggests activation of the HIF pathway, direct confirmation of HIF-1α protein stabilization (e.g., by Western blotting or immunohistochemical staining) was not performed. This prevents us from concluding with certainty that the observed neuroprotective and behavioral effects are mediated specifically by HIF-1α stabilization rather than by other HIF-independent mechanisms of Roxadustat action. Quantitative assessment of HIF-1α protein levels will be a priority for future research. Additionally, evaluation of functional HIF-1 transcriptional activity using reporter systems or measurement of classical HIF-dependent target genes (VEGF, EPO, GLUT1, LDHA, and HO-1) is of considerable interest. Furthermore, investigating the effects of Roxadustat on astrocyte and microglial activation profiles represents a promising direction. Future studies should also examine the dynamics of amyloid pathology in more detail to confirm whether these pathways are modulated by Roxadustat.

It will also be necessary to study the effects of chronic Roxadustat administration in healthy wild-type animals to assess potential side effects, since the present study focused on the effects of Roxadustat on neurodegenerative processes in 5xFAD animals.

Another direction for future research should be the investigation of the effects of HIF-1 stabilization on the electrophysiological properties of neurons. Currently, data on the effects of Roxadustat on synaptic plasticity are limited and rather contradictory. In the study by Moreton et al. [22], electrophysiological experiments on rat hippocampal slices demonstrated that Roxadustat prevents neuronal death following hypoxia and oxygen–glucose deprivation (OGD), yet suppresses long-term potentiation (LTP), but exclusively under conditions of post ischemic stress (hypoxia), and not under OGD or oxidative stress induced by hydrogen peroxide. The authors suggest that this effect is associated with disrupted AMPA receptor trafficking. Additionally, it has been shown that seven-day pharmacological inhibition of PHD with DMOG, leading to HIF-1α stabilization, also suppressed LTP in the CA1 region but not in the dentate gyrus, indicating regional specificity of this effect [58]. At the end of treatment, all groups showed no change in synaptic excitability using paired pulse paradigms. On the other hand, in vivo studies in models of depression induced by chronic stress or lipopolysaccharide [59] and in aging models demonstrate that Roxadustat improves cognitive functions, enhances synaptic plasticity, and promotes dendritic growth through HIF 1 dependent activation of the CREB/BDNF and PI3K signaling pathways. Therefore, conducting electrophysiological studies in the future is of great interest.

## 5. Conclusions

A four-week course of Roxadustat administration exerted a positive effect on neurological status and learning ability in male 5xFAD mice. In addition, a reduction in pathological morphological alterations in the cerebral cortex was observed. These findings suggest that the neuroprotective effects associated with HIF-1 stabilization are mediated, at least in part, by increased expression of the neurotrophic factor BDNF, though a causal relationship requires further confirmation.

## Figures and Tables

**Table 1:** Primers used in the study.

Gene	Primer Sequences	Amplicon Lengths, b.p.
*Oaz1*	fw 5′-AAGGACAGTTTTGCAGCTCTCC-3′ rv 5′-TCTGTCCTCACGGTTCTTGGG-3′	93
*App*	fw 5′-ATGCAGAATTCCGACATGACTCAGGA-3′ rv 5′-CACCATGAGTCCAATGATTGCACCTT-3′	107
*Bdnf*	fw 5′-CCCAACGAAGAAAACCATAAGGA-3′ rv 5′-CCAGCAGAAAGAGTAGAGGAGGCT-3′	97
*Gdnf*	fw 5′-CCTTCGCGCTGACCAGTGACT-3′ rv 5′-GCCGCTTGTTTATCTGGTGACC-3′	121
*Hif-1α*	fw 5′-GCAATTCTCCAAGCCCTCCAAG-3′ rv 5′-TTCATCAGTGGTGGCAGTTGTG-3′	110
*Il-1β*	fw 5′-GCCCATCCTCTGTGACTCATGG-3′ rv 5′-GTTCATCTCGGAGCCTGTAGTGC-3′	92
*Il-6*	fw 5′-AGTCCGGAGAGGAGACTTCACAGAG-3′ rv 5′-CACGATTTCCCAGAGAACATGTGTAA-3′	103
*Tnf-α*	fw 5′-GCCCACGTCGTAGCAAACC-3′ rv 5′-TGGTTGTCTTTGAGATCCATGCC-3′	101

**Table 2:** Morphometric characteristics of prefrontal cortex tissue in mouse brains.

Parameter	WT	5xFAD	5xFAD + Roxadustat
Maximum cell diameter, µm	12.19 ± 0.23	13.82 ± 0.3 *	12.67 ± 0.22 #
Minimum cell diameter, µm	10.42 ± 0.2	12.06 ± 0.24 *	11.16 ± 0.19 * #
Cytoplasmic diameter, µm	2.73 ± 0.13	3.25 ± 0.18	2.55 ± 0.12 #
Nuclear diameter, µm	9.46 ± 0.18	10.56 ± 0.21 *	10.11 ± 0.18 *
Nuclear-to-cytoplasmic ratio	0.78 ± 0.01	0.77 ± 0.01	0.8 ± 0.01
Hyperchromatic neurons	6.71 ± 0.77	6.96 ± 0.96	4.62 ± 0.65
Shrunken hyperchromatic neurons	3.75 ± 0.55	5.04 ± 0.63	4.07 ± 0.55
Cells with pericellular edema	3.2 ± 0.52	5.11 ± 0.71 *	3.31 ± 0.41
Cells exhibiting signs of swelling	0.44 ± 0.1	1.73 ± 0.17 *	0.87 ± 0.14 #
Cells with cytoplasmic vacuolization	0.27 ± 0.12	1.23 ± 0.22 *	0.7 ± 0.18
Ghost cells	0.93 ± 0.14	3.96 ± 0.21 *	2.49 ± 0.18 * #
Cells exhibiting signs of apoptosis or necrosis	0.53 ± 0.11	3.24 ± 0.23 *	1.96 ± 0.19 * #
Mean number of neurons per field of view	61.04 ± 2.31	58.24 ± 2.33	60.82 ±2.36
Mean number of glial cells per field of view	3.76 ± 0.3	8.24 ± 0.61 *	6.02 ± 0.43 *

* Differences are statistically significant relative to the WT (*p* ≤ 0.05, ANOVA); # differences are statistically significant relative to the 5xFAD group (*p* ≤ 0.05, ANOVA).

## Data Availability

The data used to support the findings of this study are available from the corresponding author upon request.

## Abbreviations

The following abbreviations are used in this manuscript:

AD	Alzheimer’s disease
AICD	APP intracellular domain
APP	amyloid precursor protein
Aβ	amyloid-β
BDNF	brain-derived neurotrophic factor
EPO	erythropoietin
FIH	asparaginyl hydroxylase
GDNF	glial cell line-derived neurotrophic factor
GLUT 1	glucose transporter 1
GLUT 3	glucose transporter 3
HIF	hypoxia-inducible factor
HO-1	heme oxygenase-1
HRE	hypoxia-response element
NFT	neurofibrillary tangles
O-GlcNAc	O-linked N-acetylglucosamine
PHD	prolyl hydroxylase
pVHL	von Hippel-Lindau protein
ROS	reactive oxygen species
RT-PCR	real-time polymerase chain reaction
VEGF A	vascular endothelial growth factor A
